# Investigation into the Effect of Atmospheric Particulate Matter (PM_2.5_ and PM_10_) Concentrations on GPS Signals

**DOI:** 10.3390/s17030508

**Published:** 2017-03-03

**Authors:** Lawrence Lau, Jun He

**Affiliations:** 1 Department of Civil Engineering, The University of Nottingham Ningbo China, 199 Taikang East Road, Ningbo 315100, China; 2 Department of Chemical and Environmental Engineering, The University of Nottingham Ningbo China, 199 Taikang East Road, Ningbo 315100, China; jun.he@nottingham.edu.cn

**Keywords:** Global Positioning System (GPS), GPS signal propagation, atmospheric particulate matter (PM), PM_2.5_, PM_10_, air pollution

## Abstract

The Global Positioning System (GPS) has been widely used in navigation, surveying, geophysical and geodynamic studies, machine guidance, etc. High-precision GPS applications such as geodetic surveying need millimeter and centimeter level accuracy. Since GPS signals are affected by atmospheric effects, methods of correcting or eliminating ionospheric and tropospheric bias are needed in GPS data processing. Relative positioning can be used to mitigate the atmospheric effect, but its efficiency depends on the baseline lengths. Air pollution is a serious problem globally, especially in developing countries that causes health problems to humans and damage to the ecosystem. Respirable suspended particles are coarse particles with a diameter of 10 micrometers or less, also known as PM_10_. Moreover, fine particles with a diameter of 2.5 micrometers or less are known as PM_2.5_. GPS signals travel through the atmosphere before arriving at receivers on the Earth’s surface, and the research question posed in this paper is: are GPS signals affected by the increased concentration of the PM_2.5_/PM_10_ particles? There is no standard model of the effect of PM_2.5_/PM_10_ particles on GPS signals in GPS data processing, although an approximate generic model of non-gaseous atmospheric constituents (<1 mm) can be found in the literature. This paper investigates the effect of the concentration of PM_2.5_/PM_10_ particles on GPS signals and validates the aforementioned approximate model with a carrier-to-noise ratio (CNR)-based empirical method. Both the approximate model and the empirical results show that the atmospheric PM_2.5_/PM_10_ particles and their concentrations have a negligible effect on GPS signals and the effect is comparable with the noise level of GPS measurements.

## 1. Introduction

The Global Satellite Navigation System (GNSS) has been widely used in vehicle and personal navigation, engineering and geodetic surveying, geophysical and geodynamic studies, machine guidance, attitude determination [1], indoor positioning (with high-sensitivity receiver), etc. High-precision GNSS applications such as engineering and geodetic surveying need millimeter and centimeter level accuracy. GNSS includes the US Global Positioning System (GPS), the Russian GLObal NAvigation Satellite System (GLONASS), the European Galileo, and the Chinese Beidou (BDS); systems with global positioning coverage are considered as GNSS in this paper—BDS’s Geostationary Orbit (GEO) and Inclined Geosynchronous Orbit (IGSO) satellites are considered as Regional Navigation Satellite System (RNSS) in this paper because they only provide regional positioning capacity. GNSS signals transmit from Medium Earth Orbit (MEO) satellites passing through the space and atmosphere, and arrive at GNSS receivers on the Earth’s surface. Since GNSS signals are affected by atmospheric biases, method for correcting or eliminating any ionospheric and tropospheric bias are needed in GNSS data processing. The ionosphere is a dispersive medium, which causes different delays/advancements in measurements in different GNSS frequencies. The original GPS was designed to have two frequencies in the L1 and L2 bands, and the two frequencies can be linearly combined to eliminate the first-order ionospheric effect. All GNSS, therefore, have two or more frequencies to tackle the ionospheric effect. Moreover, the ionospheric effect can be mitigated by applying correction models such as the Klobuchar model [2] and the NeQuick model [3]. Tropospheric refraction affects all GNSS frequencies the same, i.e., the delays in different frequencies are the same. Correction models such as the Hopfield model [4], and the Saastamoinen model [5] can be used to mitigate tropospheric bias. More details about GNSS ionospheric and tropospheric effects and their correction/mitigation can be found in [6].

**Figure 1:**
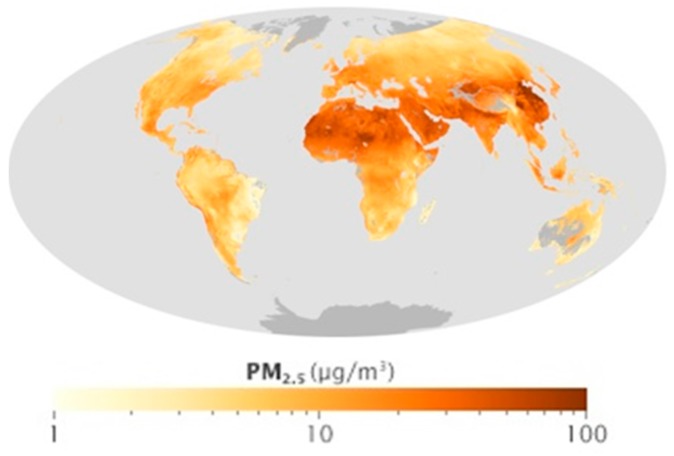
Global view of PM_2.5_ in 2001–2006 based on data from the Multi-angle Imaging SpectroRadiometer (MISR) and the Moderate Resolution Imaging Spectroradiometer (MODIS) onTerra and on the GEOS-Chem model. (Source: http://visibleearth.nasa.gov/view.php?id=46823; Credit: NASA map by Robert Simmon, based on data from the Multi-angle Imaging SpectroRadiometer (MISR) and the Moderate Resolution Imaging Spectroradiometer (MODIS) on Terra and on the GEOS-Chem model. Caption by Holli Riebeek and Adam Voiland).

**Figure 2:**
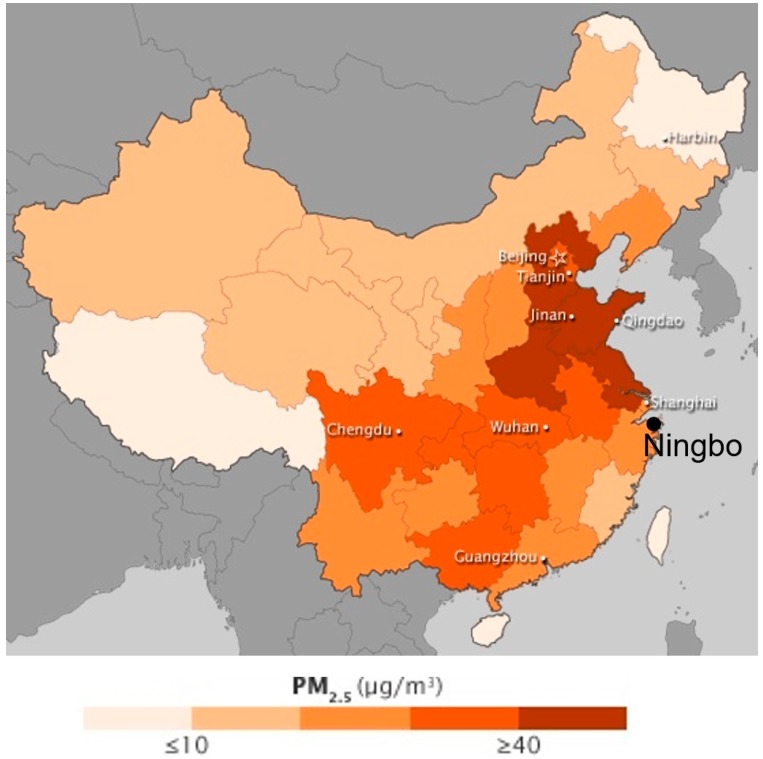
Satellite map of PM_2.5_ over China in 2008–2010 based on data from the MISR instrument on the Terra satellite, the MODIS instrument on the Terra and Aqua satellites, and a chemical transport model called GEOS-Chem. (Source: http://earthobservatory.nasa.gov/IOTD/view.php?id=77495; Credit: NASA Earth Observatory image by Jesse Allen, using data provided by Erica Zell, Battelle and Angel Hsu, Yale Center for Environmental Law & Policy. Caption by Adam Voiland.).

Air pollution is a serious environmental problem globally, especially in developing countries. Major pollutants include sulfur oxides (SO_x_), nitrogen oxides (NOx), carbon monoxide (CO), volatile organic compounds (VOC), particulates, alternatively referred to as particulate matter (PM), chlorofluorocarbons (CFCs), ammonia (NH_3_), radioactive pollutants, etc. Particulates are tiny solid or liquid particles suspended in a gas. Respirable suspended particles (RSP) are relatively coarse particles with a diameter of 10 micrometers or less, also known as PM_10_. Fine particles with a diameter of 2.5 micrometers or less is known as PM_2.5_. High levels of PM_2.5_ in the air are linked to health hazards such as heart disease [7], reduced lung function and lung cancer. A global view of PM_2.5_ density observed by the Multi-angle Imaging SpectroRadiometer (MISR) and the Moderate Resolution Imaging Spectroradiometer (MODIS) remote sensing satellites in 2001–2006 presented by NASA is shown in Figure 1. It shows PM_2.5_ density is usually high in developing countries. Figure 2 shows the PM_2.5_ density observed by the MISR and MODIS remote sensing satellites over China in 2008–2010. Ningbo, for which the location in China is shown on the map (Figure 2), is the city where test data collection took place for this investigation.

GNSS can be used to estimate the zenith tropospheric delay [8] and to estimate precipitable water vapor content [9]. GNSS signals travel through the atmosphere, and so it may be asked whether GNSS signals are refracted by the atmospheric particulate matters or not. There is no standard model of the atmospheric PM_2.5_/PM_10_ particles on GNSS signals in GNSS data processing, and the authors could only find an approximate generic model of non-gaseous atmospheric constituents (<1 mm) [10] in the literature. This paper investigates the effect of the concentration of PM_2.5_/PM_10_ particles on GPS signals and validates the aforementioned approximate generic model with a carrier-to-noise ratio (CNR) based empirical method. Note that GPS is used in this investigation because it is the only GNSS providing global coverage with code division multiple access (CDMA) signals and that is in its full operational capacity (FOC) currently. The frequency division multiple access (FDMA) based GLONASS is not used in this investigation because physical interaction is usually frequency dependent. Moreover, the long constellation geometry repeatability (i.e., 8 days) of GLONASS would cause greater meteorological uncertainty in the proposed empirical method of this paper.

The objective of this paper is to investigate any effect of the concentration of atmospheric PM_2.5_ and PM_10_ on GPS signals based on single-station GPS dual-frequency data collected on the roof of the Science and Engineering Building (SEB) at the University of Nottingham Ningbo China (UNNC). This paper is divided into five sections: Section 1 presents the introduction and background of this research. Section 2 describes briefly the physics relevant to GNSS atmospheric refraction, and the approximate generic model of non-gaseous atmospheric constituents. The methodology of this investigation and the experimental data description are given in Section 3. Processing results and analysis are presented in Section 4. Finally, concluding remarks are given in Section 5.

## 2. Physics of Atmospheric Refraction in GNSS

According to the laws of reflection and refraction, when a plane wave falls on to a boundary between two homogeneous media of different optical properties, it is split into two waves—a transmitted wave proceeding into the second medium, and a reflected wave propagated back into the first medium [11]. In GNSS, it is well known that refraction occurs when GNSS signals propagate in the ionosphere and troposphere, and reflection occurs when there is multipath [12,13].

The ionospheric effects on the phase (*δ_p_*) and code (*δ_g_*) signal transmission path (*s*) can be represented as [6]:

(1) $$\delta_{p}=\int \left(n_{p}-1\right)ds=\int \left(\frac{a_{1}}{f^{2}}+\frac{a_{2}}{f^{3}}\right)ds$$

where *n_p_* denotes the refractive index of ionosphere on phase, coefficients *a*_1_ and *a*_2_ depend on the electronic density *N_e_*, and *f* is the frequency:

(2) $$\delta_{g}=\int \left(n_{g}-1\right)ds=\int \left(-\frac{a_{1}}{f^{2}}-\frac{a_{2}}{f^{3}}\right)ds$$

where *n_g_* denotes the refractive index of ionosphere on group. Omitting the second term on the right-hand side of Equations (1) and (2), we get:

(3) $$\delta_{p}=-\delta_{g}=\int \left(\frac{a_{1}}{f^{2}}\right)ds$$

The ionospheric effects on the phase and code measurements have the opposite signs and have approximately the same magnitude. The coefficient *a*_1_ is −40.3*N_e_*, where *N_e_* is the electronic density. The total electronic content (TEC) in the zenith direction can be defined as:

(4) $$TEC=\int N_{e}ds$$

which can be computed from special models; see [6] for the details. Particulate matters are trapped in the planetary boundary layer that is usually below 2 km measured from sea level [14] while the ionosphere is in the region of 50–1500 km measured from sea level [15]. Therefore, no physical interaction between particulate matters in the lower atmosphere with ions/electrons in the ionosphere is possible.

Tropospheric effect depends on the temperature, pressure, humidity and altitude of the antenna location, it causes the same delays in different GNSS frequencies and measurement types (i.e., pseudorange and carrier phase). Similar to the ionospheric path delay, the tropospheric path delay can be written as [6]:

(5) δ=∫(n−1)ds

where *n* is the refractive index of the troposphere, the integration is taken along the signal path. Scaling of the refractive index anomaly (*n* − 1) is usually made by:

(6) $$N=10^{6}\left(n-1\right)$$

where *N* is called tropospheric refractivity. *N* can be divided into the wet (about 10%) and dry (about 90%) components:

(7) $$N=N_{w}+N_{d}$$

where indices *w* and *d* denote the wet and dry components, which are caused by the water vapour and dry atmosphere, respectively. Equation (5) becomes:

(8) $$\delta =\delta_{w}+\delta_{d}=10^{-6}\int Nds$$

where the wet component of the tropospheric path delay (*δ_w_*):

(9) $$\delta_{w}=10^{-6}\int N_{w}ds$$

and, the dry component of the tropospheric path delay (*δ_d_*):

(10) $$\delta_{d}=10^{-6}\int N_{d}ds$$

Particulate matter (PM_2.5_ and PM_10_) may float in the air/gas or dissolve in water and become an aerosol [16], suspended in the troposphere [17]. The presence of particulate matter may change the refractivity of the troposphere. When an electromagnetic wave propagates through the “layer” of particulate matter, the possible physical interactions are reflection, refraction, absorption and scattering. GNSS frequency range (about 1.2–1.6 GHz) can be considered as very short radio waves or very long microwaves in the electromagnetic spectrum. We know that we can still receive GNSS signals when there is PM_2.5_/PM_10_ particulate matter in the atmosphere, therefore, the most likely and important physical interaction is refraction. However, it could be that the PM_2.5_/PM_10_ particulate matter has no physical effect on GNSS signals. If the refractive index of the particle is only slightly different than that of the surrounding medium [18]:

(11) |n−1|≪1

The unity inside the absolute value symbol in Equation (11) is the refractive index of the medium relative to itself, because *n* is the refractive index of the particle relative to that of the medium. If the condition expressed by Equation (11) is fulfilled, then the particle interacts very weakly with the incident light [18]; this may not be applicable to other electromagnetic frequencies. Nephelometry is used to measure the aerosol scattering coefficient, and it is suggested that PM_2.5_ has the real part of refractive index in the range of 1.3–1.8 and the imaginary part of refractive index in the range of 0.000–0.200 in the visible light spectrum [19]; the wavelength in the visible light spectrum is shorter than the diameter of particulate matter and it is much shorter than the GNSS signal wavelengths. We know that a large variety of chemical compounds are involved in the formation of aerosols, and at the same time it is extremely difficult to determine the chemical composition of an atmospheric aerosol. A lack of accurate knowledge of this chemical composition makes it difficult to infer the property that strongly depends upon it, namely, the refractive index (both real and imaginary) [20]; in the literature a value of 1.55 has often been assigned for atmospheric aerosols [21]. The imaginary refractive index of atmospheric particulate matter in the 0.3–1.7 µm spectral region shows seasonal and geographic variations [22]; note that this spectrum is much shorter than the GNSS spectrum and the imaginary refraction (absorption) is not a concern in this paper. In practice, most refractivity measurement methods such as a specular reflection technique of normal incidence at the surface of disks made out of aerosol and Abbe’s refractometer could cause chemical and/or physical changes and thus result in an error in the estimation of the in situ refractive index [21].

There is no physical model of the effect of particulate matter PM_2.5_/PM_10_ on GNSS signals in the literature. An approximate generic model of non-gaseous atmospheric constituents (<1 mm), based on the Clausius-Mossotti equation for refractivity, is given in [10] as:

(12) $$\left(n-1\right)\times 10^{6}=N=1.5\times 10^{6}\frac{M}{\rho }\left[\frac{\varepsilon -1}{\varepsilon +2}\right]$$

where *M* denotes the mass content of the particles per unit of air volume, *ρ* denotes the density of the particles, *ɛ* denotes the permittivity of the particles, and M/ρ is the mass fraction of the suspended particles.

As stated above, due to the complex composition of particulate matter and the difficulties of measuring the refractive index of the particulate matter in the atmosphere, it is difficult to assess the impact of particulate matter (PM_2.5_/PM_10_) and its concentration on the tropospheric refractive index and on the GNSS signal propagation. This paper uses GPS raw carrier-to-noise ratio (CNR) data in consecutive sidereal days to analyze the impact of particulate matter on GNSS signal propagation, and validate the approximate generic model Equation (12).

## 3. Methodology of Investigation and Experimental Data Description

Since the receiver clock offset of a GNSS receiver is not a constant, we cannot compare GNSS pseudorange and carrier-phase measurements collected in high and low PM periods directly. Moreover, relative positioning techniques cannot be used to detect the impact of particulate matter on GNSS signal propagation because the assumption of different residuals due to particulate matter in different atmospheric paths cannot be justified. An empirical method is used to investigate any impact of particulate matter on GNSS signal propagation, and the detail is described as follows.

**Figure 3:**
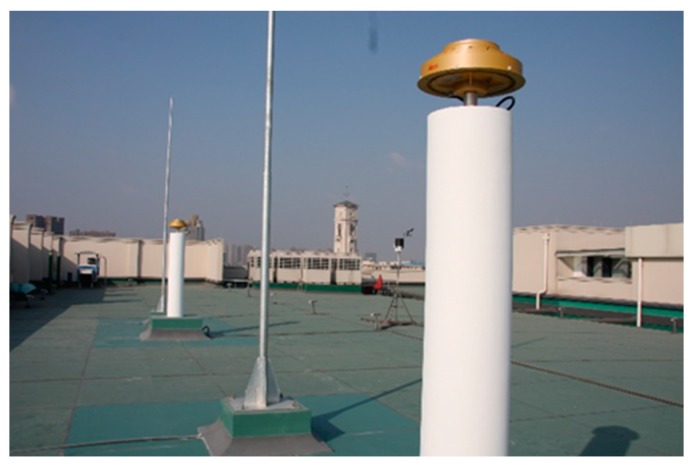
GNSS reference stations in UNNC.

The empirical method is to compare the CNR data in the unit of dBHz in two consecutive sidereal days with similar and very different PM indices; it is called the CNR method, see below. Owing to the unique condition of repeatable satellite geometry in about one sidereal day at continuous static antennas, the same multipath errors repeat at the same sidereal time of the next day [13] and so as the CNR if there are no abnormal ionospheric and tropospheric activities such as ionospheric scintillation. The method of calculating sidereal days used in this paper can be found in [23]. Six GPS data sets collected at a GNSS reference station (with a Leica GR25 receiver and AT20 antenna) on the roof of the Science and Engineering Building (SEB) at the University of Nottingham Ningbo China (UNNC) are used in this investigation, and PM_10_, PM_2.5_ and Air Quality Index (AQI) data was collected at a national air quality monitoring station located at the Ningbo Wanli University, which is about 1.5 km from UNNC and the altitudes of the two stations are similar (about 20 m). Details of PM_10_, PM_2.5_, AQI indices and their conversion to concentration of pollutant in µg/m^3^ can be found in [24]; for example, PM_2.5_ AQI 50 = 12 µg/m^3^, PM_2.5_ AQI 200 = 150.4 µg/m^3^, PM_2.5_ AQI 300 = 250.4 µg/m^3^, PM_10_ AQI 50 = 54 µg/m^3^, PM_10_ AQI 200 = 354 µg/m^3^, PM_10_ AQI 300 = 424 µg/m^3^. The UNNC GNSS reference stations on the roof are shown in Figure 3. Two selected data sets were collected in November 2014 and four data sets were collected in December 2015, the values of PM_10_, PM_2.5_ and AQI indices of the six data sets are shown in Table 1, Table 2, Table 3 and Table 4. Periods of high (>200) and low (<50) PM indices are selected for this investigation, ratios of PM_10_, PM_2.5_ and AQI indices between the same time periods of the first day and second day are also shown in the tables. Data set 1 has great differences in PM_10_, PM_2.5_ and AQI indices between the two sidereal days (6–8 November 2014), and the ratios of the indices are in the range of about 3 to 12 as shown in the last three columns of Table 1. On the other hand, the differences in PM_10_, PM_2.5_ and AQI indices between the two sidereal days (7–9 November 2014) in Data set 2 are small, the ratios of the indices are in the range of about 0.6 to 1.8 (mainly around 1) as shown in the last three columns of Table 2. Data sets 3–5 are the data sets with large differences in PM_10_, PM_2.5_ and AQI indices in two sidereal days (15–17, 23–25, 23–24 in December 2015), the ratios are about 4 as shown in Table 3, Table 4 and Table 5. Data set 6 has small differences in PM_10_, PM_2.5_ and AQI indices in the two sidereal days in 27–29 December 2015, and the ratios are close to 1 as shown in Table 6. If there is an impact of particulate matter and its concentration on GNSS signal propagation such as refraction, the CNR should be different when the ratios of PM_10_, PM_2.5_ and AQI indices in two sidereal days are large. Data sets (i.e., Data sets 2 and 6) with low ratios (~1) of PM_10_, PM_2.5_ and AQI indices in two sidereal days are used to show the nominal differences in CNR. If there is no noticeable change in CNR (<the nominal CNR difference) when the PM_10_, PM_2.5_ and AQI ratios between sidereal days are large, then particulate matter and its concentration may have no or very insignificant impact on GNSS signal propagation. The maximum AQI index in the scale is 500 [24], the maximum AQI index in the data sets is 314 while the minimum AQI index is 20. The quality of the GPS data sets was checked using the Translate/Edit/Quality Check (TEQC) software [25], and no abnormal data is found in the data sets. Moreover, the temperatures, pressures and relative humidity values between the sidereal days in the data sets are similar, the effect of the small differences of them translated to tropospheric delay is analyzed in the next section.

## 4. Results, Analysis and Discussion

### 4.1. Description of Results

**Figure 4:**
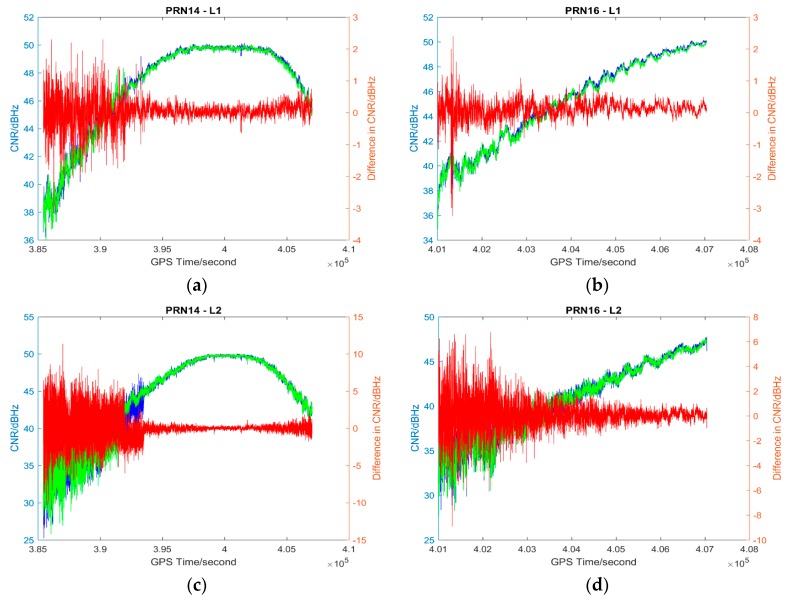
Plots of L1 (**a**,**b**) and L2 (**c**,**d**) CNR of satellites PRN14 and PRN16 in Day 1 vs. L1 and L2 CNR of the satellites in Day 2 of Data set 1; Blue: Day 1 CNR, Green: Day 2 CNR, Red: the differences in Day 1 and Day 2 CNR.

**Figure 5:**
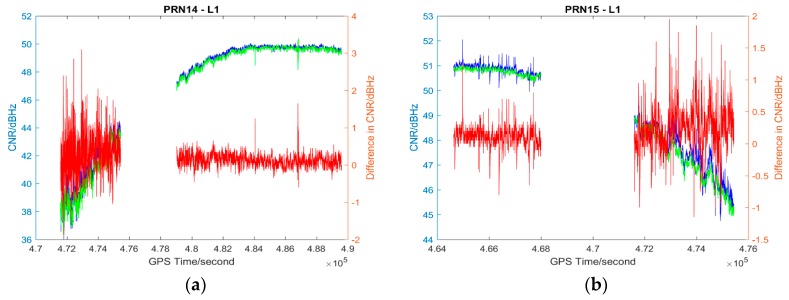

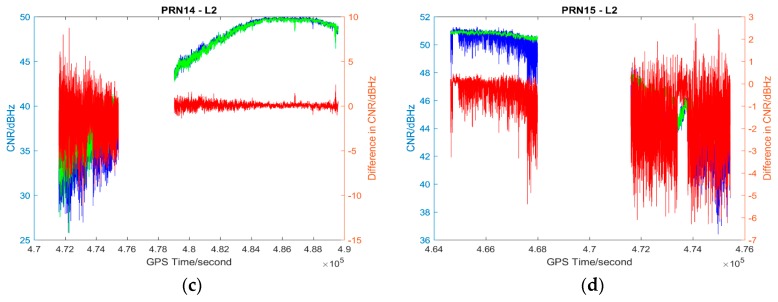
Plots of L1 (**a**,**b**) and L2 (**c**,**d**) CNR of satellites PRN14 and PRN15 in Day 1 vs. L1 and L2 CNR of the satellites in Day 2 of Data set 2; Blue: Day 1 CNR, Green: Day 2 CNR, Red: the differences in Day 1 and Day 2 CNR; data gaps in the plots are due to a receiver data logging error.

**Figure 6:**
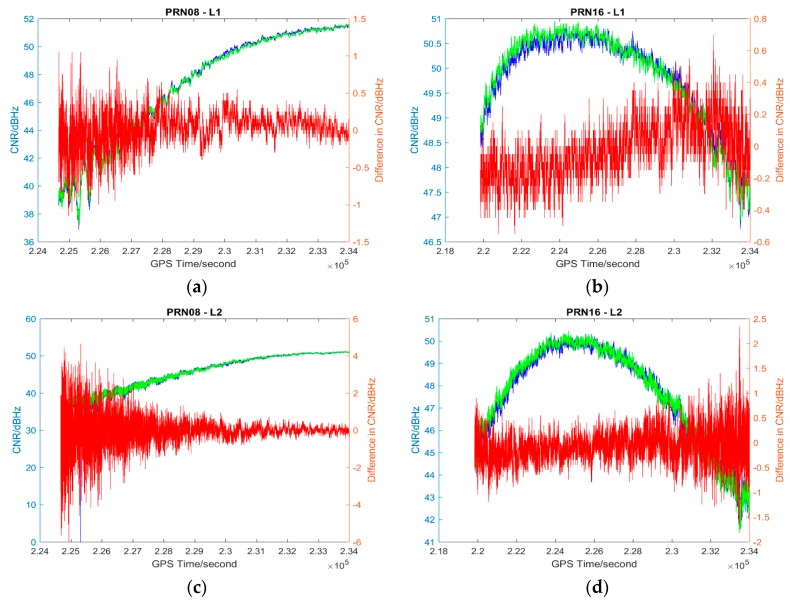
Plots of L1 (**a**,**b**) and L2 (**c**,**d**) CNR of satellites PRN08 and PRN16 in Day 1 vs. L1 and L2 CNR of the satellites in Day 2 of Data set 3; Blue: Day 1 CNR, Green: Day 2 CNR, Red: the differences in Day 1 and Day 2 CNR.

**Figure 7:**
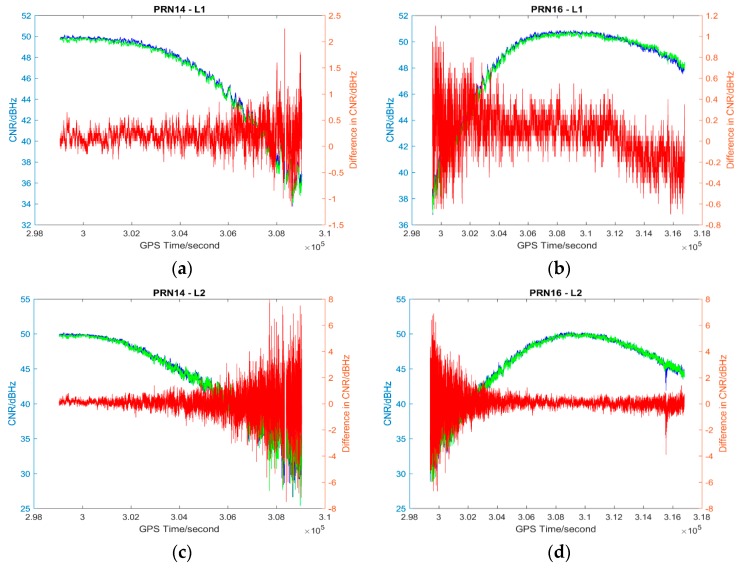
Plots of L1 (**a**,**b**) and L2 (**c**,**d**) CNR of satellites PRN14 and PRN16 in Day 1 vs. L1 and L2 CNR of the satellites in Day 2 of Data set 4; Blue: Day 1 CNR, Green: Day 2 CNR, Red: the differences in Day 1 and Day 2 CNR.

**Figure 8:**
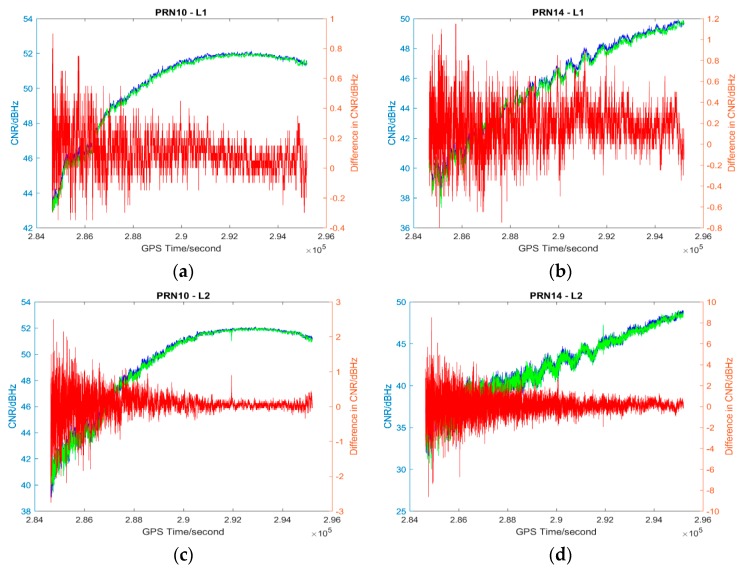
Plots of L1 (**a**,**b**) and L2 (**c**,**d**) CNR of satellites PRN10 and PRN14 in Day 1 vs. L1 and L2 CNR of the satellites in Day 2 of Data set 5; Blue: Day 1 CNR, Green: Day 2 CNR, Red: the differences in Day 1 and Day 2 CNR.

**Figure 9:**
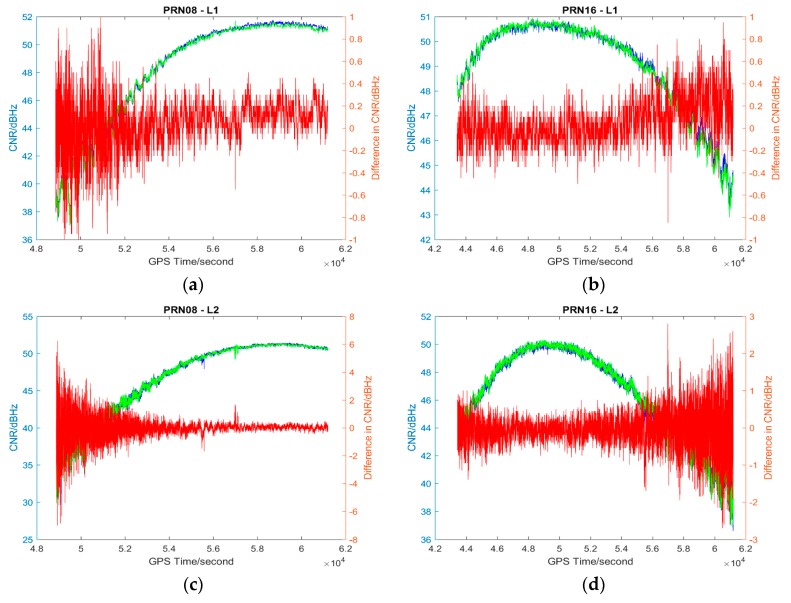
Plots of L1 (**a**,**b**) and L2 (**c**,**d**) CNR of satellites PRN08 and PRN16 in Day 1 vs. L1 and L2 CNR of the satellites in Day 2 of Data set 6; Blue: Day 1 CNR, Green: Day 2 CNR, Red: the differences in Day 1 and Day 2 CNR.

The L1 and L2 CNR and their differences on two consecutive sidereal days of the selected satellites in the six data sets are shown in Figure 4, Figure 5, Figure 6, Figure 7, Figure 8 and Figure 9; only two satellites per data set are shown due to the page limit. Moreover, the statistical results (mean and standard deviation (S.D.) in 95% confidence level) of the L1 and L2 CNR differences (i.e., Day 1 CNR–Day 2 CNR) in two consecutive sidereal days with the satellite elevation angle greater than 30° are presented in Table 7 and Table 8. The 30° satellite elevation mask is used in order to reduce the effect of the high noise level at low elevation angles on the statistical results.

### 4.2. Analysis of Results

The CNR differences of some satellites are positive and some are negative with positive being the majority in Data set 1 (see Table 7), we cannot see that the high PM_10_, PM_2.5_ and AQI indices in Day 1 (see Table 1) always lead to a reduction of CNR. Day 1 CNR minus Day 2 CNR would be always negative if the particulate matter had an impact on the GNSS signal propagation, which would lead to a reduction in signal strength and an increase in the noise level. In no physical and chemical circumstances would the additional particulate matter in the atmosphere increase the GNSS CNR. The overall mean values of L1 and L2 CNR differences of Data set 1 are less than those of Data set 2 (see Table 7, absolute values are considered). Since Data set 1 has great differences in PM_10_, PM_2.5_ and AQI indices between sidereal days (see Table 1) while Data set 2 has similar PM_10_, PM_2.5_ and AQI indices between sidereal days (see Table 2), we cannot find any significant impact of particulate matter on GNSS signal propagation based on the results of the data sets collected in November 2014.

Data sets 3–5 have great differences in PM_10_, PM_2.5_ and AQI indices between consecutive sidereal days (see Table 3, Table 4 and Table 5) while Data set 6 has similar PM_10_, PM_2.5_ and AQI indices between consecutive sidereal days (see Table 4). Similar to the data sets collected in November 2014, the CNR differences of some satellites are positive and some are negative with positive as the majority in Data sets 3–5 collected in December 2015 (see Table 8). The CNR differences in Data set 6 are less than the CNR differences in Data sets 3–5; however, the standard deviations are similar (see Table 8). Nevertheless, all the mean values of CNR differences in Data sets 1, 3–5 are much less than 1 dBHz, which is much less significant as the multipath effect [26].

The effect of the different temperatures, pressures and relative humidity values on tropospheric delays between sidereal days in the data sets is estimated. The hourly meteorological data collected at the national air quality monitoring station at Wanli University was put in the Saastamoinen tropospheric model [5,6], and the computed ZTDs in Days 1 and 2 and their differences of the six data sets are shown in Table 9. It shows that the effect of the different temperatures, pressures and relative humidity values on tropospheric delays between sidereal days is very small, therefore, the effect of the different meteorological conditions on CNR is negligible.

When we put a very high mass fraction of PM_2.5_/PM_10_ particles as 0.3 [27] and the relative permittivity of PM_2.5_ as 30 and PM_10_ as 3.8 [28] in (12), the path delays of PM_2.5_ and PM_10_ particles are about 0.2 and 0.4 mm, respectively, if 1 km is assumed as the PM_2.5_/PM_10_ layer height. When a simple mapping function of 1/cos(zenith angle of satellite) is applied, the path delays of PM_2.5_ and PM_10_ particles become about 1.3 and 2.4 mm, respectively, when the zenith angle of a satellite is 80° (i.e., the elevation angle is 10°). Therefore, based on (12), the path delays induced by PM_2.5_/PM_10_ particles are in the similar range of GPS carrier-phase measurement noise or slightly larger when compared with the GNSS carrier-phase measurement noise levels described in [29].

## 5. Conclusions

The Global Satellite Navigation System (GNSS) has been widely used in vehicle and personal navigation, engineering and geodetic surveying, geophysical and geodynamic studies, machine guidance, attitude determination, indoor positioning (with high-sensitivity receiver), etc. High-precision GNSS applications such as engineering and geodetic surveying need millimeter and centimeter level accuracy. Air pollution is a serious environmental problem globally, especially in developing countries. Major pollutants include sulfur oxides, nitrogen oxides, carbon monoxide, volatile organic compounds, particulate matter (PM), chlorofluorocarbons, ammonia, radioactive pollutants, etc. GPS signals travel through the atmosphere before arriving at receivers on the Earth’s surface, but are GPS signals affected by the increased concentration of the PM_2.5_/PM_10_ particles? There is no standard model of the effect of PM_2.5_/PM_10_ particles on GNSS signals in GPS data processing, although an approximate generic model of non-gaseous atmospheric constituents (<1 mm) can be found in the literature. Through knowing the unique condition of repeatable satellite geometry of GPS in about one sidereal day at continuous static antennas, this paper presented an empirical method to investigate the impact of PM_2.5_/PM_10_ particles and their concentrations on GPS signal propagation. The method was used to compare the carrier-to-noise ratios (CNRs) in two consecutive sidereal days with similar and very different PM indices. Six data sets were collected on the campus of the University of Nottingham Ningbo China in November 2014 and December 2015 for this investigation. Since the receiver clock offset of a GNSS receiver is not a constant, we cannot compare GNSS pseudorange and carrier-phase measurements collected in high and low PM periods directly. Moreover, relative positioning techniques cannot be used to detect the impact of particulate matter on GNSS signal propagation because the assumption of differenced residuals due to particulate matter in different atmospheric paths cannot be justified. CNR is not affected by the different receiver clock offsets at different measurement epochs and it is not affected by multipath effect when the same sidereal time is used in the comparison. Therefore, CNR is used in this investigation.

In the CNR test, the results are mixed, but with more negative results than positive results (i.e., reduced CNR due to the presence of PM_2.5_/PM_10_ particles). All the CNR differences are much less than 1 dBHz, which is much less significant than the multipath effect. The CNR differences between consecutive sidereal days are likely due to the GPS carrier-phase measurement noise. Therefore, we cannot find any significant impact of high PM_2.5_/PM_10_ concentration on GPS signal propagation with the presented empirical method and the six data sets collected. This result agrees with the computed delays from the approximate generic model.

## Figures and Tables

**Table 1:** PM_10_, PM_2.5_ and AQI indices and their ratios between the two sidereal days of Data set 1.

Date	Time (hh:mm)	PM_10_	PM_2.5_	AQI	PM_10_ Ratio	PM_2.5_ Ratio	AQI Ratio
6 November 2014	19:00	144	222	222	4.0	9.7	6.2
6 November 2014	20:00	130	203	203	3.9	9.2	6.2
6 November 2014	21:00	130	203	203	4.2	10.2	6.5
6 November 2014	22:00	142	216	216	4.3	9.8	6.5
6 November 2014	23:00	148	223	223	4.9	11.7	7.4
7 November 2014	00:00	147	221	221	4.7	11.1	7.1
7 November 2014	01:00	141	213	213	4.1	9.3	6.3
7 November 2014	02:00	136	207	207	3.0	6.9	4.6
7 November 2014	19:00	36	23	36	-	-	-
7 November 2014	20:00	33	22	33	-	-	-
7 November 2014	21:00	31	20	31	-	-	-
7 November 2014	22:00	33	22	33	-	-	-
7 November 2014	23:00	30	19	30	-	-	-
8 November 2014	00:00	31	20	31	-	-	-
8 November 2014	01:00	34	23	34	-	-	-
8 November 2014	02:00	45	30	45			

**Table 2:** PM_10_, PM_2.5_ and AQI indices and their ratios between the two sidereal days of Data set 2.

Date	Time (hh:mm)	PM_10_	PM_2.5_	AQI	PM_10_ Ratio	PM_2.5_ Ratio	AQI Ratio
7 November 2014	16:00	38	26	38	0.9	0.7	0.9
7 November 2014	17:00	32	22	32	1.0	0.7	0.9
7 November 2014	18:00	30	19	30	0.9	0.6	0.9
7 November 2014	19:00	36	23	36	1.1	0.7	1.0
7 November 2014	20:00	33	22	33	1.0	0.7	1.0
7 November 2014	21:00	31	20	31	1.3	0.8	1.2
7 November 2014	22:00	33	22	33	1.8	1.1	1.7
7 November 2014	23:00	30	19	30	1.7	1.1	1.5
8 November 2014	00:00	31	20	31	1.3	0.9	1.3
8 November 2014	16:00	42	39	42	-	-	-
8 November 2014	17:00	32	30	36	-	-	-
8 November 2014	18:00	33	32	35	-	-	-
8 November 2014	19:00	34	35	35	-	-	-
8 November 2014	20:00	33	33	33	-	-	-
8 November 2014	21:00	23	26	26	-	-	-
8 November 2014	22:00	18	20	20	-	-	-
8 November 2014	23:00	18	18	20	-	-	-
9 November 2014	00:00	24	23	24	-	-	-

**Table 3:** PM_10_, PM_2.5_ and AQI indices and their ratios between the two sidereal days of Data set 3.

Date	Time (hh:mm)	PM_10_	PM_2.5_	AQI	PM_10_ Ratio	PM_2.5_ Ratio	AQI Ratio
15 December 2015	21:00	181	280	280	4.2	9.3	6.5
15 December 2015	22:00	191	295	295	4.0	8.9	6.1
15 December 2015	23:00	194	298	298	3.7	7.8	5.6
16 December 2015	00:00	192	293	293	3.6	7.0	5.4
16 December 2015	01:00	189	290	290	3.4	6.3	5.2
16 December 2015	21:00	43	30	43			
16 December 2015	22:00	48	33	48			
16 December 2015	23:00	53	38	53			
17 December 2015	00:00	54	42	54			
17 December 2015	01:00	56	46	56			

**Table 4:** PM_10_, PM_2.5_ and AQI indices and their ratios between the two sidereal days of Data set 4.

Date	Time (hh:mm)	PM_10_	PM_2.5_	AQI	PM_10_ Ratio	PM_2.5_ Ratio	AQI Ratio
23 December 2015	19:00	200	314	314	4.9	6.0	6.0
23 December 2015	20:00	194	305	305	4.7	6.1	6.1
23 December 2015	21:00	189	295	295	4.5	5.7	5.7
23 December 2015	22:00	190	293	293	3.9	5.0	5.0
23 December 2015	23:00	191	294	294	3.7	4.7	4.7
24 December 2015	00:00	194	294	294	3.7	4.7	4.7
24 December 2015	19:00	41	52	52			
24 December 2015	20:00	41	50	50			
24 December 2015	21:00	42	52	52			
24 December 2015	22:00	49	59	59			
24 December 2015	23:00	51	62	62			
25 December 2015	00:00	52	63	63			

**Table 5:** PM_10_, PM_2.5_ and AQI indices and their ratios between the two sidereal days of Data set 5.

Date	Time (hh:mm)	PM_10_	PM_2.5_	AQI	PM_10_ Ratio	PM_2.5_ Ratio	AQI Ratio
23 December 2015	15:00	136	221	221	4.0	5.3	5.3
23 December 2015	16:00	143	229	229	3.3	4.2	4.2
23 December 2015	17:00	153	240	240	3.8	4.6	4.6
23 December 2015	18:00	166	258	258	4.2	5.0	5.0
24 December 2015	15:00	34	42	42			
24 December 2015	16:00	44	55	55			
24 December 2015	17:00	40	52	52			
24 December 2015	18:00	40	52	52			

**Table 6:** PM_10_, PM_2.5_ and AQI indices and their ratios between the two sidereal days of Data set 6.

Date	Time (hh:mm)	PM_10_	PM_2.5_	AQI	PM_10_ Ratio	PM_2.5_ Ratio	AQI Ratio
27 December 2015	20:00	55	48	55	0.7	0.6	0.7
27 December 2015	21:00	55	49	55	0.7	0.7	0.7
27 December 2015	22:00	56	50	56	0.7	0.6	0.7
27 December 2015	23:00	57	53	57	0.8	0.8	0.8
28 December 2015	00:00	56	50	56	0.8	0.7	0.7
28 December 2015	01:00	54	48	54	0.7	0.6	0.7
28 December 2015	20:00	75	74	75			
28 December 2015	21:00	75	75	75			
28 December 2015	22:00	76	78	78			
28 December 2015	23:00	68	67	68			
29 December 2015	00:00	74	75	75			
29 December 2015	01:00	75	79	79			

**Table 7:** 95% confidence level statistical results of the L1 and L2 CNR differences of selected satellites in two consecutive sidereal days in the Datasets 1 and 2 collected in November 2014. Data with the satellite elevation angle greater than 30° is used.

Data Set 1 Great AQI Difference	L1 (dBHz)		L2 (dBHz)	
PRN	Mean	S.D.	Mean	S.D.
14	0.059	0.160	−0.056	0.560
16	0.159	0.145	0.051	0.371
18	0.016	0.191	0.011	0.809
21	−0.017	0.317	0.345	1.523
24	−0.009	0.247	0.100	1.074
Overall	0.042		0.090	
**Data set 2 Similar AQI**	**L1 (dBHz)**		**L2 (dBHz)**	
PRN	Mean	S.D.	Mean	S.D.
14	0.124	0.127	0.079	0.221
15	0.184	0.196	−0.975	1.085
18	0.097	0.144	−0.043	0.386
24	0.157	0.167	−0.702	0.744
29	0.167	0.191	0.072	0.857
Overall	0.146		−0.314	

**Table 8:** 95% confidence level statistical results of the L1 and L2 CNR differences of selected satellites in two consecutive sidereal days in the Datasets 3 to 6 collected in December 2015. Data with the satellite elevation angle greater than 30° is used.

Data Set 3 Great AQI Diff	L1 (dBHz)		L2 (dBHz)	
PRN	Mean	S.D.	Mean	S.D.
08	0.077	0.125	−0.012	0.257
16	−0.029	0.1556	−0.075	0.251
26	−0.047	0.166	−0.087	0.230
27	0.003	0.127	0.015	0.156
Overall	0.001		−0.039	
**Data set 4 Great AQI diff**	**L1 (dBHz)**		**L2 (dBHz)**	
PRN	Mean	S.D.	Mean	S.D.
14	0.185	0.132	0.094	0.314
16	0.049	0.173	0.052	0.263
26	0.084	0.162	0.079	0.215
27	−0.019	0.152	−0.050	0.176
Overall	0.075		0.044	
**Data set 5 Great AQI diff**	**L1 (dBHz)**		**L2 (dBHz)**	
	Mean	S.D.	Mean	S.D.
10	0.092	0.097	0.072	0.171
12	0.168	0.147	0.101	0.346
14	0.193	0.147	0.108	0.414
18	0.088	0.109	0.058	0.205
Overall	0.135		0.085	
**Data set 6 Similar AQI**	**L1 (dBHz)**		**L2 (dBHz)**	
	Mean	S.D.	Mean	S.D.
08	0.077	0.125	0.013	0.220
16	0.041	0.150	−0.041	0.286
26	−0.019	0.121	−0.041	0.209
27	0.050	0.124	0.025	0.164
Overall	0.037		−0.011	

**Table 9:** Saastamoinen modelled ZTD in Day 1 and Day 2 and their differences of the six data sets.

Data Set 1	Day 1 ZTD (m)	Day 2 ZTD (m)	ΔZTD (m)
19:00	2.460	2.459	0.0008
20:00	2.466	2.460	0.0063
21:00	2.468	2.462	0.0059
22:00	2.470	2.460	0.0103
23:00	2.471	2.471	−0.0007
00:00	2.468	2.470	−0.0027
01:00	2.466	2.475	−0.0090
02:00	2.464	2.482	−0.0179
		mean	−0.0009
**Data set 2**	**Day 1 ZTD (m)**	**Day 2 ZTD (m)**	**ΔZTD (m)**
16:00	2.455	2.492	−0.0364
17:00	2.457	2.493	−0.0356
18:00	2.459	2.492	−0.0332
19:00	2.459	2.493	−0.0335
20:00	2.460	2.492	−0.0322
21:00	2.462	2.491	−0.0281
22:00	2.460	2.489	−0.0294
23:00	2.471	2.484	−0.0122
00:00	2.470	2.482	−0.0112
		mean	−0.0280
**Data set 3**	**Day 1 ZTD (m)**	**Day 2 ZTD (m)**	**ΔZTD (m)**
21:00	2.417	2.388	0.0284
22:00	2.419	2.389	0.0296
23:00	2.419	2.386	0.0329
00:00	2.418	2.385	0.0324
01:00	2.418	2.389	0.0286
		mean	0.0304
**Data set 4**	**Day 1 ZTD (m)**	**Day 2 ZTD (m)**	**ΔZTD (m)**
19:00	2.440	2.448	−0.0085
20:00	2.439	2.446	−0.0070
21:00	2.442	2.448	−0.0052
22:00	2.444	2.448	−0.0041
23:00	2.443	2.445	−0.0017
00:00	2.444	2.444	0.0002
		mean	−0.0044
**Data set 5**	**Day 1 ZTD (m)**	**Day 2 ZTD (m)**	**ΔZTD (m)**
15:00	2.437	2.451	−0.0145
16:00	2.441	2.452	−0.0114
17:00	2.443	2.450	−0.0072
18:00	2.442	2.451	−0.0088
		mean	−0.0105
**Data set 6**	**Day 1 ZTD (m)**	**Day 2 ZTD (m)**	**ΔZTD (m)**
20:00	2.450	2.424	0.0260
21:00	2.446	2.420	0.0265
22:00	2.446	2.422	0.0239
23:00	2.442	2.422	0.0203
00:00	2.442	2.416	0.0260
01:00	2.442	2.415	0.0270
		mean	0.0250
